# Mixing with native broadleaf trees modified soil microbial communities of *Cunninghamia lanceolata* monocultures in South China

**DOI:** 10.3389/fmicb.2024.1372128

**Published:** 2024-03-05

**Authors:** Fenglin Zheng, Jiawei Gu, Dehao Lu, Jiaman Yang, Xiaomai Shuai, Cheng Li, Hongyue Chen

**Affiliations:** College of Forestry and Landscape Architecture, South China Agricultural University, Guangzhou, China

**Keywords:** pure *Cunninghamia lanceolata* forest, mixed *Cunninghamia lanceolata*-broadleaf forest, soil microbial diversity, microbial community structure, microbial symbiotic network

## Abstract

Mixing with different broadleaf trees into the monocultures of *Cunninghamia lanceolata* is widely adopted as an efficient transformation of the pure *C*. *lanceolata* forest. However, it is unclear how native broad-leaved trees influence the belowground ecological environment of the pure *C*. *lanceolata* culture plantation in nutrient-poor soil of South China. Herein, we aimed to investigate how a long-time mixing with native broadleaf trees shape soil microbial community of the pure *C. lanceolata* forest across different soil depth (0–20 cm and 20–40 cm) and to clarify relationships between the modified soil microbial community and those affected soil chemical properties. Using high-throughput sequencing technology, microbial compositions from the mixed *C. lanceolata*-broadleaf forest and the pure *C. lanceolata* forest were analyzed. Network analysis was utilized to investigate correlations among microorganisms, and network robustness was assessed by calculating network natural connectivity. Results demonstrated that the content of soil microbial biomass carbon and nitrogen, total phosphorus and pH in mixed forest stand were significantly higher than those in pure forest stand, except for available phosphorus in topsoil (0–20 cm). Simultaneously, the mixed *C. lanceolata*-broadleaf forest has a more homogeneous bacterial and fungal communities across different soil depth compared with the pure *C. lanceolata* forest, wherein the mixed forest recruited more diverse bacterial community in subsoil (20–40 cm) and reduced the diversity of fungal community in topsoil. Meanwhile, the mixed forest showed higher bacterial community stability while the pure forest showed higher fungal community stability. Moreover, bacterial communities showed significant correlations with various soil chemical indicators, whereas fungal communities exhibited correlations with only TP and pH. Therefore, the mixed *C. lanceolata*-broadleaf forest rely on their recruiting bacterial community to enhance and maintain the higher nutrient status of soil while the pure *C. lanceolata* forest rely on some specific fungi to satisfy their phosphorus requirement for survive strategy.

## Introduction

*Cunninghamia lanceolata*, indigenous to China, is a fast-growing commercial timber species. Recognized for its swift growth, top-notch timber, and substantial yield, the monoculture of *C. lanceolata* offers noteworthy economic benefits. However, the monoculture of *C. lanceolata* has given rise to an array of issues, such as soil degradation, diminished growth of trees, and an increased incidence of pests and diseases, due to a lack of biodiversity (Yang et al., 2019). Consequently, there has been a noticeable decline in soil quality, fertility, and ecosystem stability in the pure *C. lanceolata* plantations, severely affecting their productivity and the sustainability of their management (Chauvat et al., 2011; Wang et al., 2014). To alleviate these adverse effects of monoculture forests, the practice of intermixing with broad-leaved tree species has been widely adopted in recent years. Establishing mixed forests comprising *C. lanceolata* and selected broad-leaved tree species not only ameliorates soil degradation and reductions in stand productivity but also enhances the stability of forest ecosystems (Liu et al., 2018). Wang et al. (2009) found that the introduction of broad-leaved species into pure *C. lanceolata* forest increases biomass carbon and soil carbon storage in the mixed forest. The improvement of soil fertility in mixed forests correlates with alterations in litter fall and soil microbial activity (Chauvat et al., 2011). Broad-leaved species produce more litter than conifers, and their litter decomposes more rapidly, accelerating the nutrient returns from litterfall to the soil and thus improving soil fertility (Trum et al., 2011). Furthermore, mixed forests can enhance ecological benefits by promoting niche differentiation among different species (Lian et al., 2022). Yang et al. (2023) suggests that the mixed plantation can heighten the overall ecological benefits of afforestation, potentially fortifying forests against extreme climate events. In forest ecosystems, different forest stand patterns not only influence the physical and chemical properties of the soil but also exhibit a close association with soil microbial communities (Gui et al., 2022; Tian et al., 2023).

Soil microbes, serving as the primary decomposer, play a pivotal role in directing energy flow within soil ecosystems and contribute significantly to essential ecological processes, including nutrient cycling, organic matter decomposition, and biological nitrogen fixation (King, 2014). Plants, soil, and microbes form a dynamically balanced ecosystem essential for the growth and health of plants (Philippot et al., 2013). Among interactions between soil microbes and plants, soil microbial community would be shaped by exudates secreted by plant roots (Haichar et al., 2014), which consist of soluble carbohydrates, amino acids, organic acids, hormones, antibiotics, enzymes, and other compounds. These substances serve as nutritional sources and growth factors for microbes, promoting microbial metabolism and interactions between microbes and plants (Berg et al., 2013). Various factors, including plant species, environmental conditions, and physiological status of plant, regulate the composition and quantity of these secretions (Chaparro et al., 2014). Among soil microbes, bacteria play a crucial role in forest ecosystems, which inhabit various habitats including rhizosphere, deciduous leaves, and dead wood (Lladó et al., 2017). Soil bacterial community is involved in some key soil processes, such as carbon, nitrogen, and phosphorus cycle, while nutrient availability also influence the composition of soil bacterial community (Yu et al., 2021; Zhang et al., 2021). Moreover, some bacteria are associated with the production or absorption of specific nutrients, such as Rhizobia being one of several group of bacteria capable of 'fixing' nitrogen (Maitra et al., 2023), phosphate-solubilizing bacteria having the potential to make insoluble phosphate available to plants through solubilization and mineralization (Cheng et al., 2023). As for soil fungi, they also play a crucial role in forest ecosystems, such as forming symbiotic relationships with trees to enhance nutrient uptake of host plants, decomposing litterfall, and interacting with plant roots through a saprotrophic nutritional model to facilitate nutrient uptake and metabolic activities of trees (Uroz et al., 2016). Research conducted by Xu et al. (2021) on the microbial communities in mixed forests of *C. lanceolata* and *Pinus bournei* indicated that soil fungal diversity and all indices related to nutrient cycle increased with the age of the mixed forest. As for the soil depth, Guo et al. (2021) indicated that afforestation practices have significant impacts on the soil bacterial community, especially in the topsoil where variation in nutrient level respond more sensitive to land conversions (Jobbágy and Jackson, 2004). Recent studies have compared pure *C. lanceolata* plantations with mixed *C. lanceolata*-broadleaf plantations, primarily focusing on soil physical and chemical properties and biological characteristics. Bu et al. (2020) demonstrates that the mixed *C. lanceolata*-broadleaf forest can enhance soil quality, particularly in terms of chemical properties. Other studies indicate that the mixed forest may enhance soil phosphorus availability through rhizosphere effects, wherein it may lead to reduced phosphorus and nitrogen concentrations in *C. lanceolata* tissues due to competition with certain broadleaf tree species (Zhou et al., 2020). On the contrary, Yu et al. (2019) found that the introduction of broadleaved tree species could store greater amounts of SOC than single-species *C. lanceolata*. However, the microecological environment following establishment of mixed *C. lanceolata*-broadleaf plantations, especially concerning the soil microbial community, is still unclear.

Therefore, this study aims to investigate how a long-time mixing with native broadleaf trees shape soil microbial community of the pure *C. lanceolata* forest across different soil depth (0–20 cm and 20–40 cm) and to clarify relationships between the modified soil microbial community and those affected soil chemical properties. We tested the hypothesis that the long-time mixing with native broadleaf trees would enhance the soil nutrient level with the higher diversity of soil microbial community. Moreover, we tested the hypothesis that the ecological environment of the topsoil (0–20 cm) would be shaped more significantly by the transformation of *C*. *lanceolata* cultivation pattern compared with that of the subsoil (20–40 cm). Our results on variations of soil nutrient level and corresponding shaped soil microbial community, might provide an important reference for the transformation of coniferous forests into mixed coniferous and broad-leaved forests.

## Materials and methods

### Study sites and sample sites survey

The research area is located in Yunyong Forest Farm (112°38′26″-112°42′25″ E, 22°41′54″-22°46′50″ N), Foshan City, Guangdong Province, China. It has a subtropical monsoon climate with mild temperatures and abundant rainfall, averaging 23.2°C in annual temperature and ~1,900 mm in annual precipitation. The predominant soils in the research area are red soils derived from weathered granite and sandstone, with a soil depth ranging from 0.4 to 1.0 m. The humus layer is 2.5–4.0 cm thick, and the soil is mildly acidic with a pH range of 4.1–4.5. The surveyed forest stands comprise 26-year-old pure *C. lanceolata* stands and mixed *C. lanceolata*-broadleaf stands. During the initial three years, both stands underwent cutting, weeding, and fertilization. No tending measures were applied from the 4th to the 7th year. Thinning to improve light transmission and pruning occurred in the 8th year, while tending thinning was performed in the 10th to 11th years. Companion tree species in the mixed *C. lanceolata*-broadleaf stands include *Cinnamomum burmanni, Cinnamomum camphora, Michelia macclurei, Schima wallichii*, and *Castanopsis fissa*.

Sample collection was conducted in June 2023. Briefly, two types of forest stands, the pure *C. lanceolata* forest stand (PC) and the mixed *C. lanceolata*-broadleaf forest stand (MC), with the similar environmental conditions in the Yunyong Forest Farm as the surveyed forest stands. Field surveying and sampling were carried out in June 2023, documenting factors such as diameter at breast height, tree density, and topographical features like slope, aspect, and geomorphology (Table 1).

**Table 1:** Characteristics of the pure *Cunninghamia lanceolata* forest and the mixed *C. lanceolata*-broadleaf forest in surveyed forest stands.

**Forest types**	**Sample sites**	**Altitude/m**	**Slope orientations**	**Slope/°**	**Stand age/a**	**Landform types**	**Stand density/(plants·hm^−2^)**	**Average DBH/cm**
Mixed *C. lanceolata*-broadleaf plantation	1	105	Semi-sunny	22	26	Low-mountain	2,450	13.04
	2	110		26		Low-mountain	1,675	13.71
	3	115		27		Low-mountain	1,925	14.14
Pure *C. lanceolata* plantation	1	168	Semi-sunny	29	26	Low-mountain	1,450	17.68
	2	184		25		Low-mountain	1,125	17.78
	3	198		27		Low-mountain	1,050	16.46

### Soil sample collection

In June 2023, soil samples were collected from the pure *C. lanceolata* forest stand (PC) and the mixed *C. lanceolata*-broadleaf forest stand (MC) at different depths (0–20 cm, 20–40 cm). Three sites were selected in the pure *C. lanceolata* stand and mixed *C. lanceolata*-broadleaf stand. And then, a 30 m × 30 m quadrat was set up in each site. Within each quadrat, after removing litter layer, nine soil cores were collected along an S-shaped transect at ~10 m intervals using a 2.5 cm corer at different depths and composited by horizon to create three soil samples per depth range. Thus, nine soil samples were collected from PC and MC at different depths, respectively. Thus, we collected 36 soil samples in total. Soil samples were collected from 0 to 20 cm and 20 to 40 cm depths in PC and MC labeled as PC20, PC40 and MC20, MC40, respectively. Subsequently, each soil sample was divided into two portions: one portion was left to air-dry naturally for assessing soil chemical properties, and the other portion was stored at −80°C for total soil DNA extraction to analyze the composition and diversity of the soil microbial community.

### Determination of soil chemical properties

Soil microbial biomass carbon (SMBC) was determined by fumigation-extraction followed by volumetric analysis, while soil microbial biomass nitrogen (SMBN) was quantified using fumigation-extraction followed by total nitrogen determination (Liang et al., 2011). Soil pH was measured with a fresh soil/water ratio of 1:5 (vol/vol) by using an FE20 pH meter (FiveEasy; Giessen, Hessen Land, German). Soil organic carbon (SOC) was quantified by the potassium dichromate oxidation method with external heating (Nóbrega et al., 2015). Total nitrogen (TN) and total phosphorus (TP) were determined using a Kjeldahl apparatus (Qiu et al., 2023). Total potassium (TK) was measured using the sodium hydroxide fusion followed by flame photometric spectrometry. Available hydrogen nitrogen (AN) was determined by the indophenol blue colorimetric method, available phosphorus (AP) by sodium bicarbonate extraction followed by the molybdenum-antimony anti-colorimetric method (Xie et al., 2022), and available potassium (AK) by ammonium acetate extraction followed by flame photometric spectrometry (Basak, 2019).

### Soil DNA extraction and high-throughput sequencing

According to Soil Fast DNA TM SPIN kit (MP Biomedicals Co. Ltd., Santa Ana, CA, USA) kit, total soil DNA was extracted from 0.5 g fresh soil (Liu et al., 2020). After soil total DNA extraction, Nanodrop 2000 nucleic acid quantitative instrument (Thermo Scientific, Wilmington, DE, USA) was used to preliminarily detect and quantify the quality of soil total DNA (Yang et al., 2021). And then, the soil total DNA were send to the Magigene sequencing laboratory (Guangdong Magigene Biotechnology Co., Ltd.) for second-generation amplicon sequencing. Sequencing was performed using the PE250 mode of the Illumina Novaseq 6000 high-throughput sequencing platform (Chung and Kasper, 2010). During high-throughput sequencing, the V3-V4 region of the 16S rRNA gene (front-end primer sequence ACTCCTACGGGAGGCAGCAG, back-end primer sequence GGACTACHVGGGTWTCTAAT) (Tian et al., 2022) was determined for bacteria in rhizosphere soil samples to identify bacterial community diversity, and the ITS1–2 region (front-end primer sequence CTTGGTCATTTAGAGGAAGTAA, back-end primer sequence GCTGCGTTCTTCATCGATGC) (Lv et al., 2019) was determined for fungi in rhizosphere soil samples to identify fungal community diversity. Naïve Bayes Classifier (consensus_taxonomy_all_levels_99%, SILVA_132_QIIME_release) (Nilsson et al., 2019) was used to classify bacterial 16s rRNA gene sequences, and UNITE database was used to annotate fungal ITS sequences. The bacterial and fungal DNA sequences of soil samples have been deposited in BOX (https://app.box.com/s/-1gg0wzz99ekr6uuk58g2288-nn9cwhb91) and (https://app.box.com/s/vp7ni9hwk033fl-gmoruz40u4heuxkyxg), respectively.

### Statistical analysis

One-way analysis of variance (ANOVA) was used to test the significance of soil chemical properties in different soil layers of two forest stands. Trimmomatic (v0.39) was used to cut the original paired data for sequence quality control and remove adapter (Liao and Shi, 2020). According to the Barcode sequence and PCR amplification primer sequence, each sample data was separated from the off-line data, and the Barcode and primer sequences were intercepted and loaded into FLASH (v1.2.11) (Magoč and Salzberg, 2011). The reads of each sample were spliced, and the raw data obtained by splicing were subjected to strict filtering to obtain high-quality clean reads (Bokulich et al., 2013). Then the combined reads were loaded into QIIME2 (QIIME2-2023.02;qiimetoolsimport-type 'SampleData [SequencesWithQuality] ' -input-formatPairedEndFastqManifestPhred33V2) (Bolyen et al., 2019). Based on the Illumina amplicon data, DADA2 (Divisive Amplicon Denoising Algorithm) is used to denoise the amplicon data and perform double-end merging to obtain a representative sequence of single-base accuracy, generate a feature sequence and construct a feature table. Then, alpha and beta diversity of bacterial and fungal communities were calculated by using the feature table (Bolyen et al., 2019). The Vegan package (V2.6.2) of R language (V4.2) was used to calculate the alpha and beta diversity index, and the multivariate analysis of variance was performed using the Adonis function. The beta diversity of the microbial community was visualized by the principal coordinate analysis (PCoA) based on the Bray-Curtis distance. LDA Effect Size (LEfSe) Analysis (LDA > 4, *P* < 0.05) was conducted to identify microbial groups with significant differences in abundance from phylum to genus level between different groups (Segata et al., 2011). Correlation analysis of microbial communities and environmental factors was conducted according to the method described by Zhang et al. (2016), and then the correlation between the abundance of bacteria and fungi at the species level and the soil properties were conducted by Mantel's test by vegan package of R language (Guillot and Rousset, 2013), and the BrayCurtis distance between microbial communities was calculated to conduct Spearman correlation test with environmental factor matrix. Visualization was conducted using the ggplot2 and corrplot packages. In order to indicated the interaction between different phyla of soil microorganisms in different soil layers of the two forest stands, the bacterial and fungal community correlation networks were constructed, and the topological parameters in each co-occurrence network were calculated, and the species that appeared more than 80% in all samples were retained (He et al., 2017). The species level microbial communities were analyzed by using the Hmisc package in R software (Chen et al., 2017), and Spearman correlation coefficient was calculated. The correlation r and significance *P* matrix were generated, and the kinds of data |*r*| > 0.8 and *P* < 0.05 were imported into the software Gephi (0.10.1) to draw the correlation network diagram and calculate its topological properties (Ren et al., 2022). Using fastnc (https://github.com/wqssf102/fastnc) software with use of random remove nodes in a network of 1%−100% anti-destroying ability test network, stochastic simulation, 1,000 times after average natural unicom to calculate and evaluate network stability (Peng and Wu, 2016; Wu et al., 2021).

## Results

### Differences on soil chemical properties between different forest stands

As shown in Table 2, as for the different soil depth (0–20 cm, 20–40 cm), SMBC, SMBN, SOC, TN, AN, and AP in topsoil (0–20 cm) were significantly higher than those in subsoil (20–40 cm) under both forest stands (*P* < 0.05). Additionally, AK in topsoil was significantly higher than those in subsoil under PC stand (*P* < 0.05). For different forest stands, SMBC, SMBN, pH and TP in MC stand were significantly higher than those in PC stand (*P* < 0.05), while AP in PC20 was significantly higher than that in MC20 (*P* < 0.05).

**Table 2:** Soil characteristics of different soil layers in pure *Cunninghamia lanceolata* forest stand and the mixed *C. lanceolata*-broadleaf forest stand.

**Forest type and soil layers**	**SMBC (mg/kg)**	**SMBN (mg/kg)**	**pH**	**SOC (g/kg)**	**TN (g/kg)**	**TP (g/kg)**	**TK (g/kg)**	**AN (mg/kg)**	**AP (mg/kg)**	**AK (mg/kg)**
MC20	394.15 ± 39.36a	70.77 ± 7.41a	4.46 ± 0.04a	14.16 ± 1.46a	1.03 ± 0.08a	0.70 ± 0.06a	29.44 ± 1.93a	103.74 ± 5.93a	0.71 ± 0.15b	86.98 ± 13.83a
MC40	255.69 ± 23.05b	52.05 ± 4.83b	4.46 ± 0.04a	10.54 ± 0.70b	0.79 ± 0.05bc	0.75 ± 0.06a	29.32 ± 1.97a	78.80 ± 4.75b	0.29 ± 0.07c	67.71 ± 8.02a
PC20	117.75 ± 10.05c	39.99 ± 2.78bc	4.16 ± 0.03b	14.87 ± 1.00a	0.89 ± 0.06ab	0.24 ± 0.01b	30.18 ± 1.20a	98.37 ± 5.69a	1.16 ± 0.20a	74.73 ± 11.77a
PC40	132.67 ± 21.91c	35.12 ± 2.95c	4.18 ± 0.03b	9.29 ± 0.99b	0.62 ± 0.04c	0.23 ± 0.01b	30.55 ± 1.49a	71.61 ± 4.54b	0.42 ± 0.10bc	48.17 ± 6.37b
Forest type	^**^	^**^	^**^	ns	^*^	^**^	ns	ns	^*^	ns
Soil layer	^*^	^*^	ns	^**^	^**^	ns	ns	^**^	^**^	^*^
CP × SL	^*^	ns	ns	ns	ns	ns	ns	ns	ns	ns

PC20 and PC40, MC20 and MC40 stand for 0-−20 cm and 20–40 cm soil layer of pure C. lanceolata forest stand and the mixed C. lanceolata-broadleaf forest stand, respectively. Same below. Data for each condition are presented as mean ± SD. Means followed by the same letter within a column do not differ significantly by Tukey's HSD test (P < 0.05, N = 9). Significant effect of two-way ANOVA: ^*^P < 0.05, ^**^P < 0.001, ns, no significant effect.

### Differences in soil microbial composition between two different forest stands

**Figure 1:**
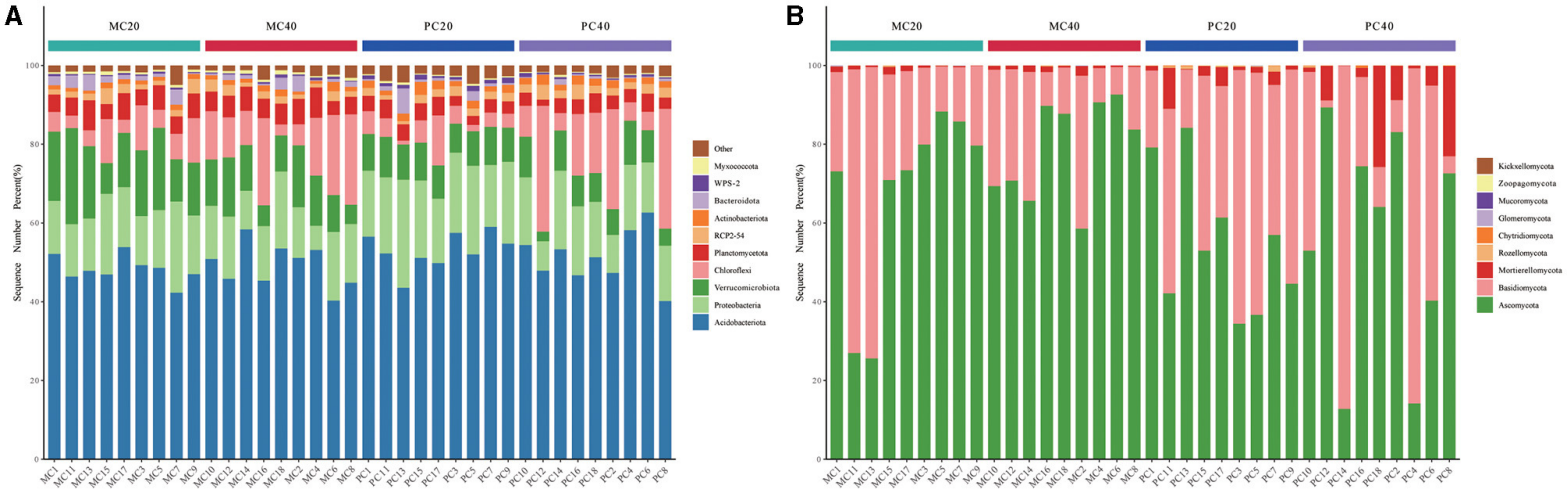
The proportion of the top 10 microorganisms in the relative abundance of bacterial **(A)** and fungal **(B)** communities under different forest stands. The abscissa is the name of the sample, and the ordinate is the proportion of species in the sample. Columns of different colors represent different genera, and the length of the column represents the proportion of genera. MC20 and MC40 represent the soil layers of 0–20 cm and 20–40 cm of the mixed *Cunninghamia lanceolata*-broadleaf forest, respectively. PC20 and PC40 represent the 0–20 cm and 20–40 cm soil layers of the pure C. lanceolata forest, respectively.

As for the total of 36 samples from MC20, MC40, PC20, and PC40 with the high-throughput sequencing technology for subsequent analysis, a total of 829 bacterial species were annotated in 36 soil samples, belonging to 42 phyla and 557 genera, a total of 360 fungal species belonging to nine phyla and 294 genera. The relative abundance of soil phylum in different soil layers of the two forest stands ranked the top 10 as shown in Figure 1A. Acidobacteriota (40.14%−62.60%) and Proteobacteria (6.16%−27.44%) were the dominant phyla under both forest stands (Figure 1A). Chloroflexi accounted for more in subsoil than in topsoil under both forest stands. Verrucomicrobiota accounted for more in MC20 than in PC20, while WPS2 accounted for more in PC20 than in MC20. Ascomycota (12.79%−92.58%) and Basidiomycota (17.67%−87.11%) were the dominant phyla in fungal community under both forest stands (Figure 1B). Mortierellomycota accounted for more in PC than MC.

**Figure 2:**
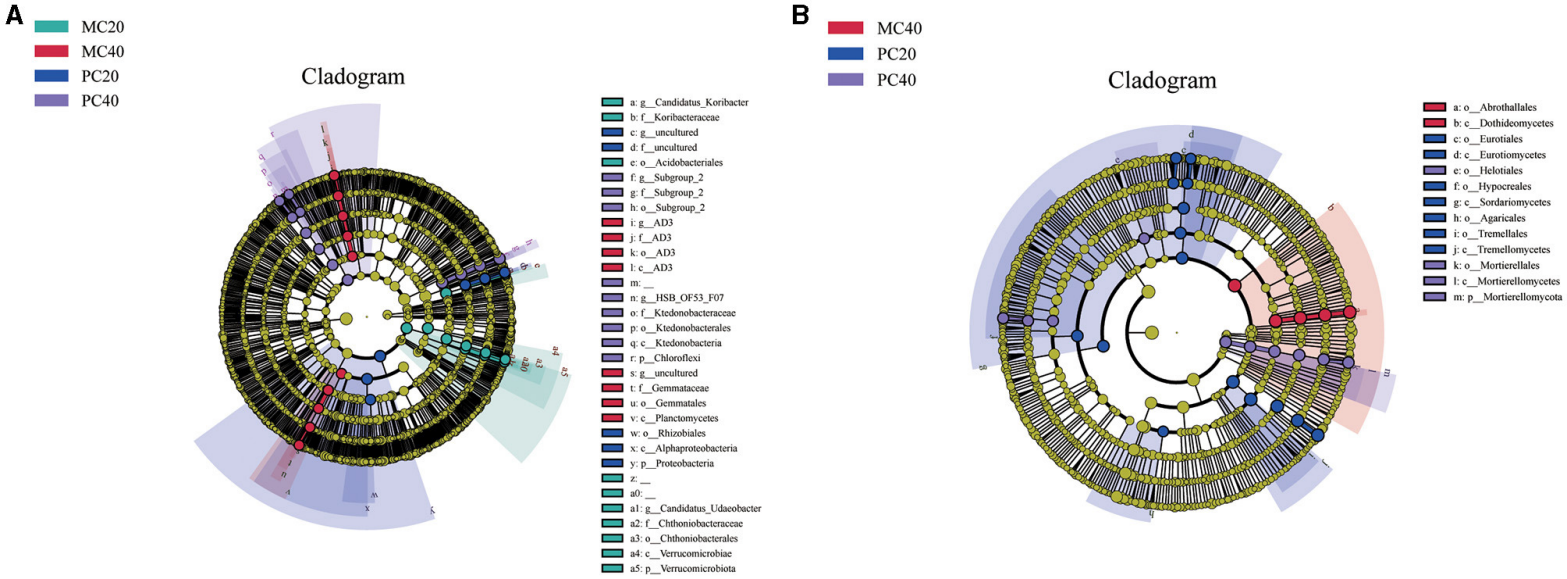
Lefse analysis of soil bacterial **(A)** and fungal **(B)** communities in different soil layers of two different stands. Significantly discriminant taxon nodes are colored, and the branch areas are shaded according to the highest ranked group for that taxon. When the taxon was not significantly differentially represented among the sample groups, the corresponding node was colored white. Highly abundant and selected taxa are indicated. Refer to Figure 1 for abbreviations of different forest stands in different soil depth.

As shown in Figure 2A, in the bacterial community, biomarkers with significant differences between MC20 and other soil samples are *Candidatus* Udaeobactera, Chthoniobacteraceaea, Chthoniobacteralesa, Verrucomicrobiaea, Verrucomicrobiota, *Candidatus* Koribacterb, Koribacteraceae, Acidobacteriales. Biomakers in MC40 samples included Gemmataceae, Gemmatales and Planctomycetes. Biomarkerss in PC20 was mainly distributed in Rhizobiales, Alphaproteobacteria and Proteobacteria, and biomarker in PC40 was mainly distributed in Ktedonobacteraceae, Ktedonobacterales, Ktedonobacteria and Chloroflexi. As for the fungal community (Figure 2B), Abrothallales and Dothideomycetes were significantly different between MC40 and other soil samples. Biomarkers in PC20 were mainly distributed in Eurotiales, Eurotiomycetes, Hypocreales, Sordariomycetes, Agaricales, Tremellales and Tremellomycetes. Biomakers in PC40 is mainly distributed in Mortierellales, Helotiales, Mortierellomycetes and Mortierellomycota. There was no significant difference between the biomarker MC20 and the other three soil samples.

### Differences in soil microbial diversity between two different forest stands

**Figure 3:**
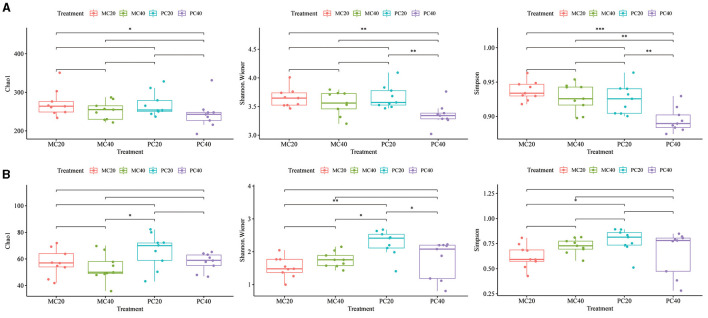
Alpha diversity of bacterial **(A)** and fungal **(B)** communities in different soil layers of different forests. The horizontal bar in the box represents the median. The top and bottom of the box represent the 75th and 25th percentiles, respectively. The asterisks above the horizontal line indicate significant differences between the two groups, *P < 0.05, **P < 0.01, and ***P < 0.001. Refer to Figure 1 for abbreviations of different forest stands in different soil depth.

The bacterial alpha diversity index (Chao1, Shannon, and Simpson index) was different among different forest types (MC and PC) in different soil depth. In Figure 3A, there was no significant difference in Chao1 index among different forest types in different soil depth (*P* > 0.05). The Shannon and Simpson index of PC20 were significantly higher than those of PC40 (*P* < 0.01), and the Simpson index of MC40 was significantly higher than that of PC40 (*P* < 0.01). The fungal alpha diversity indices (Chao1, Shannon and Simpson indices) were different among different forest types (MC and PC) in different soil depth. In Figure 3B, there was no significant difference in Chao1 index among different forest types in different soil depth (*P* > 0.05). The Shannon index of PC20 were significantly higher than those of PC40 (*P* < 0.05). The Shannon index and Simpson index of PC20 were significantly higher than those of MC20 (*P* < 0.05).

**Figure 4:**
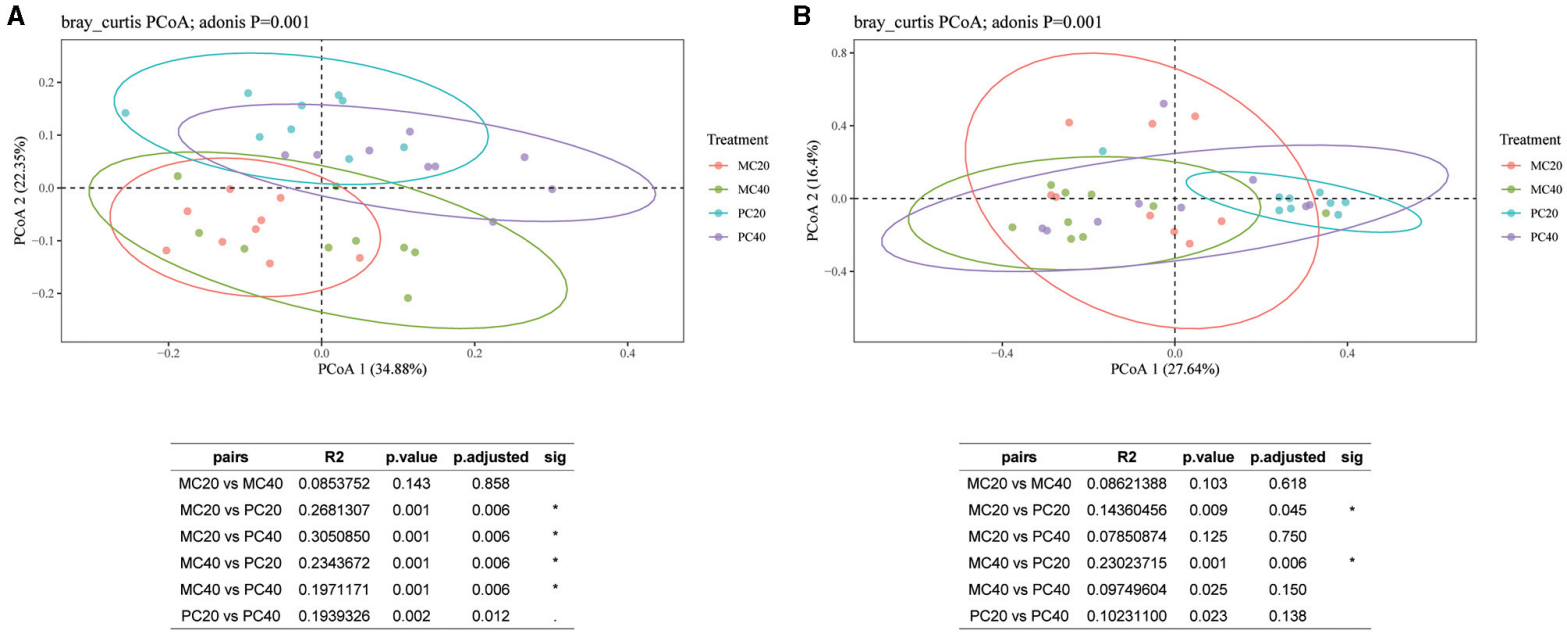
Principal coordinate analysis of different soil layers in two forest stands based on the Bray-Curtis distance. The beta diversity index was calculated by vegan package of R language, and Table **(A)** or **(B)** is the result of the multivariate analysis of variance performed using the Adonis function. *P < 0.05. Refer to Figure 1 for abbreviations of different forest stands in different soil depth.

For the bacterial communities in different soil layers of the two forest stands, the contribution of the first principal component and the second principal component were 34.88 and 22.35%, respectively (Figure 4A). As shown in Figure 4A, the confidence circles of the bacterial communities in the four treatments can be well separated, indicating that the bacterial community composition of different forest types in different soil depth is quite different. The results showed that there was no significant difference in bacterial community β diversity between MC20 and MC40 treatments (*P* > 0.05). However, there were significant differences between MC20 and PC20, MC40 and PC40, and PC20 and PC40 (*P* < 0.05, Adonis test). As for fungal community (Figure 4B), the contribution of the first principal component and the second principal component were 27.64 and 16.40%, respectively. The beta diversity of the fungal community was significantly different between MC20 and PC20, MC40 and PC40, and PC20 and PC40 (*P* < 0.05).

### Microbial co-occurrence network in two forest stands at different depths

**Figure 5:**
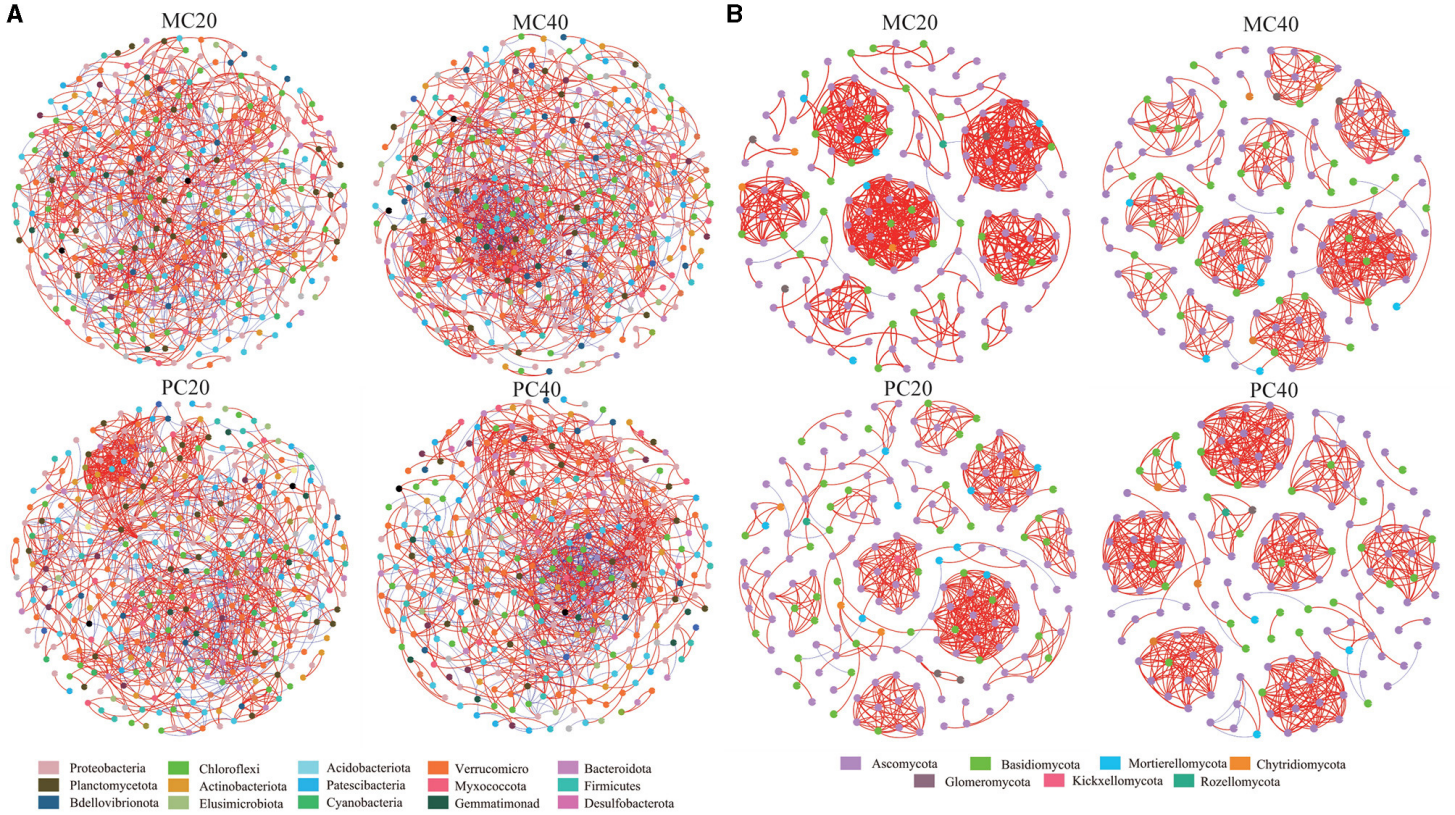
Correlation network of soil microbial bacterial **(A)** and fungal **(B)** communities in different stands and soil layers. Only the species association with extremely significant correlation was shown (|*r*| > 0.8 and *P* < 0.05), in which different nodes represent different species. The size of the node is proportional to the relative abundance of the species. Different colors represent the phylum of the species. The red connection indicates a positive correlation, the blue connection indicates a negative correlation, and the number of lines indicates the intensity of the connection between the nodes. Refer to Figure 1 for abbreviations of different forest stands in different soil depth.

The nodes in the bacterial community co-occurrence network were mainly from 15 bacterial phyla. The soil bacterial community network of MC and PC was centered on Proteobacteria and Chloroflexi (Figure 5). The co-occurrence network of topsoil was more modular than that of subsoil under both forest stands, and the average path length was higher (Table 3). The bacterial co-occurrence network of MC20 showed the lowest connectivity, while that of MC40 showed the highest connectivity. As for the fungal co-occurrence network, nodes in the fungal co-occurrence network mainly come from seven fungal phyla. Ascomycota and Basidiomycota were the core of the network of MC and PC soils (Figure 5). In contrast to the bacterial co-occurrence network, the fungal community in topsoil has a lower degree of modularity and higher average path length compared with subsoil under both forest stands, in particular for PC stand (Table 3). The fungal co-occurrence network of PC20 showed the lowest connectivity, while that of MC40 showed the highest connectivity.

**Table 3:** Topological properties of microbial co-occurrence network in different soil layers of two forests.

		**Num. edges**	**Num. vertices**	**Average. degree**	**Modularity**	**Average edge. connectivity**	**Average. path. length**	**Eigenvector centrality**
Bacterial community	MC20	372	1,131	6.081	0.667	0.385	4.674	0.051414
	MC40	364	1,448	7.956	0.634	0.469	4.465	0.026482
	PC20	381	1,347	7.071	0.699	0.448	4.542	0.065437
	PC40	323	1,247	7.721	0.611	0.435	4.526	0.024109
Fungal community	MC20	162	640	7.901	0.838	0.869	1.183	0.032833
	MC40	150	550	7.333	0.878	0.931	1.13	0.032124
	PC20	177	549	6.203	0.866	0.788	2.092	0.024918
	PC40	153	613	8.013	0.876	0.908	1.122	0.046237

**Figure 6:**
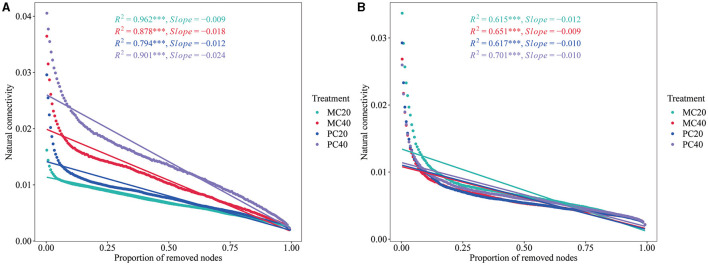
The natural connectivity of microbial networks in different soil layers of two different stands. Natural connectivity of bacterial and fungal networks was calculated and evaluated by using fastnc (https://github.com/wqssf102/fastnc) software to reveal the stability of bacterial **(A)** and fungal **(B)** networks, respectively. Refer to Figure 1 for abbreviations of different forest stands in different soil depth.

The stability of the network structure showed that the natural connectivity of bacterial networks in MC20 and PC20 showed a gentler downward trend than that in MC40 and PC40 (Figure 6A), indicating that the stability of bacterial co-occurrence network in topsoil was higher than that of subsoil. As for the different forest stands, the stability of the bacterial co-occurrence network of MC20 and MC40 was higher than that of PC20 and PC40, respectively, indicating that the soil bacterial community of MC had better anti-interference ability. In the fungal community, the opposite trend was shown (Figure 6B). By removing the same proportion of nodes, the natural connectivity of fungal networks in MC40 decreased more slowly than that of MC20, indicating that the stability of fungal co-occurrence network in subsoil was higher than that in topsoil under MC stand. As for the different stands, the stability of fungal network in PC20 was higher than that in MC20, indicating that soil fungal community in topsoil of PC had better anti-interference ability than that of MC.

### Relationship of soil microbial community with soil chemical characteristics

**Figure 7:**
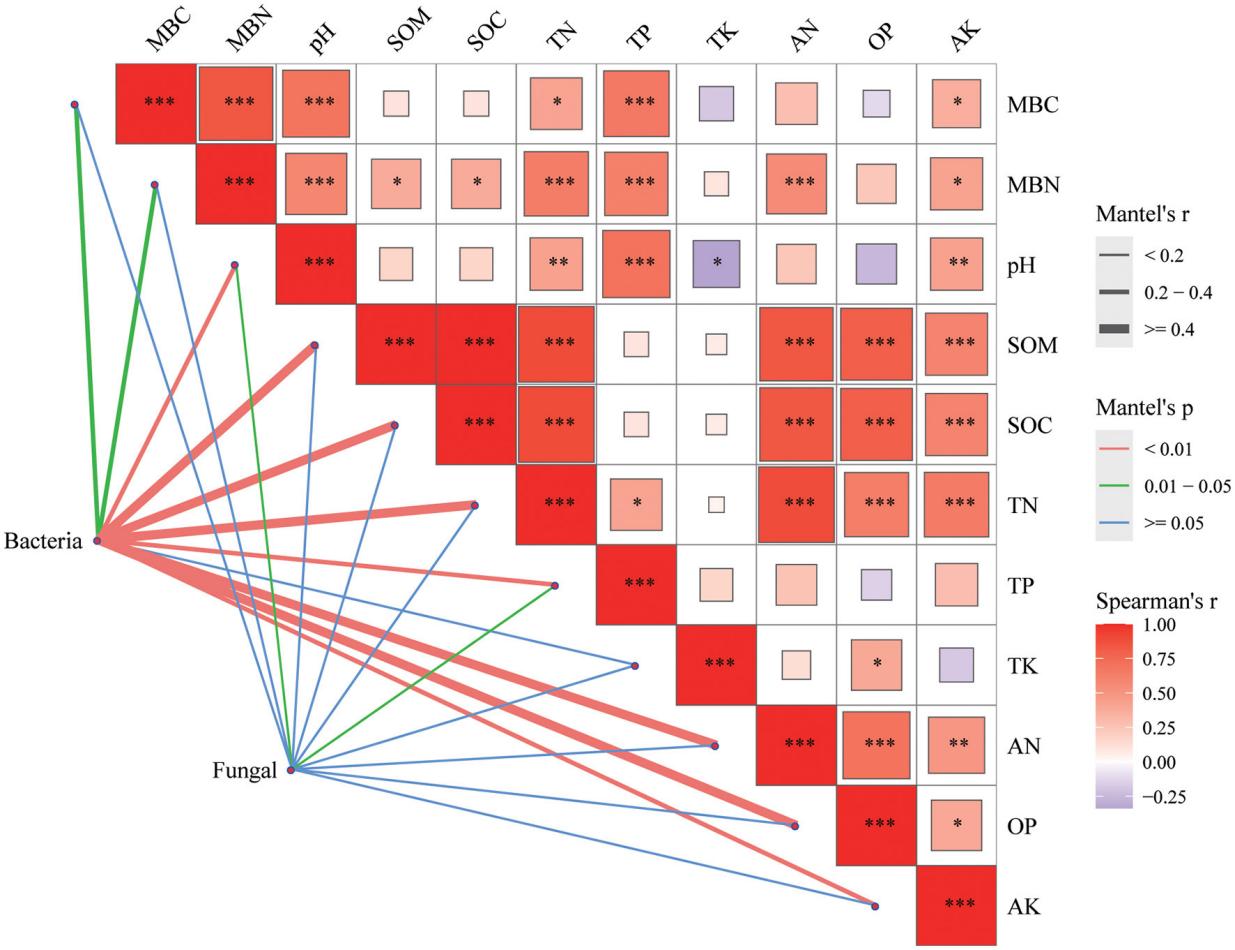
Mantel's correlation analysis between soil properties and soil microbial communities. The color of the right box represents the spearman correlation r value between environmental factors, *P < 0.05, **P < 0.01, ***P < 0.001, the left edge width corresponds to the Mantel 's r statistic of distance correlation, and the edge color represents the statistical significance *P* value based on 999 permutations.

In bacterial community, there was a significant correlation between bacterial community and MBC, MBN indexes (*P* < 0.05) and other indexes of soil properties (pH, SOM, SOC, TN, TP, AN, OP, and AK; *P* < 0.01; Figure 7). As the fungal community, in addition to the significant correlation with TP and pH (*P* < 0.05), the correlation between fungal community and other soil physical and chemical indexes was not strong.

**Figure 8:**
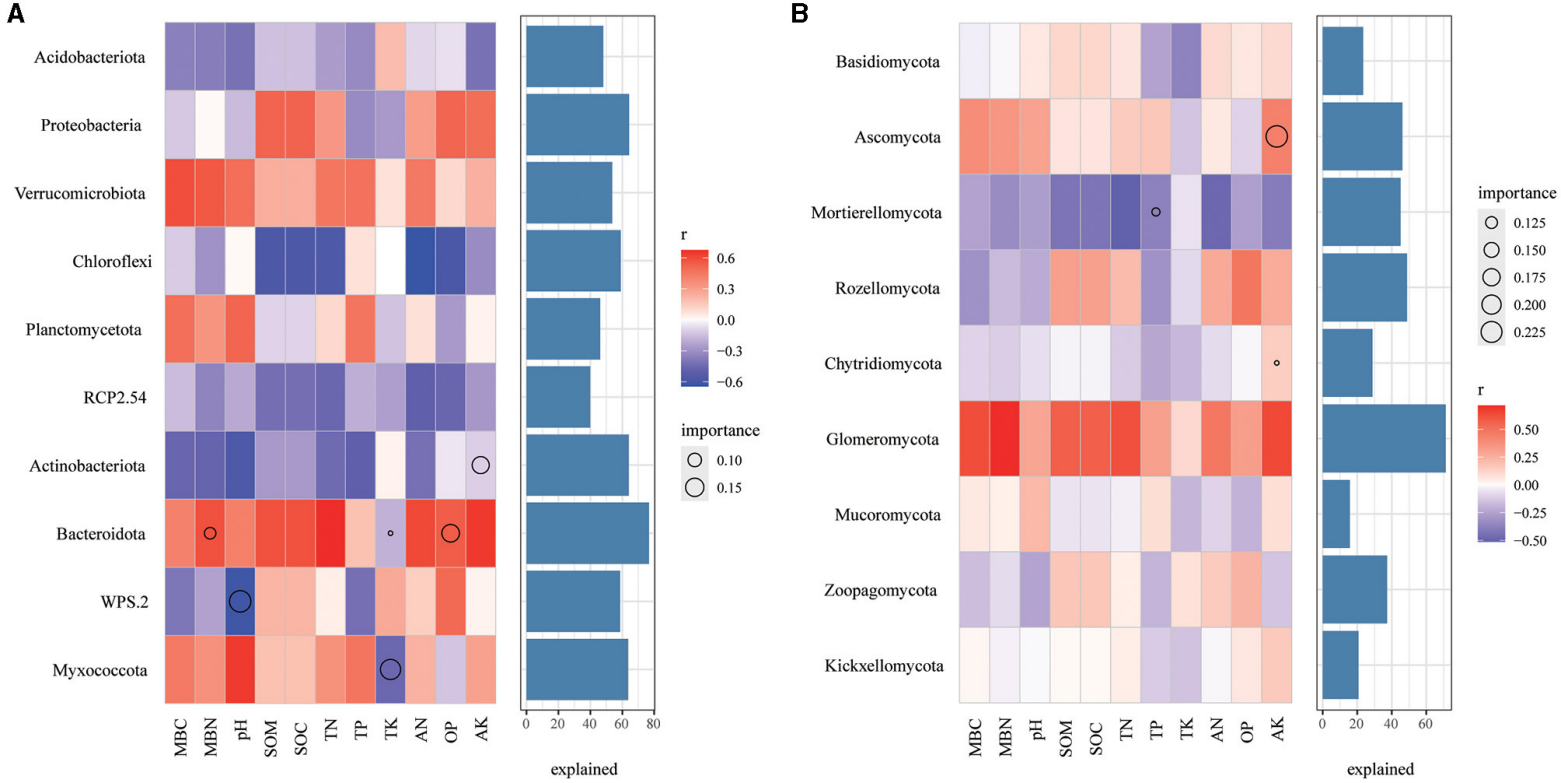
Correlations between the top 10 phyla in relative abundance and environmental factors in bacterial **(A)** and fungal **(B)** communities. The color represents the correlation coefficient, the black asterisk is the *P*-value of the correlation, the circle is the important value analyzed by multiple regression, and the bar chart on the right is the interpretation degree of environmental factors to each biological data in multiple regression.

In bacterial community, Chloroflexi were negatively correlated with SOM, SOC, TN, AN, and AP (Figure 8A), Verrucomicrobiota are positively correlated with all soil properties, while Actinobacteriota and RCP2.54 are negatively correlated with all the soil properties. Remarkably, AK, MBN and AP, pH, and TK play a significant role in the relative abundance of Actinobacteriota, Bacteroidota, WPS.2, and Myxococcota, respectively. As for the fungal community (Figure 8B), Motierellomycota was negatively correlated with almost all the soil properties, while Glomeromycota was positively correlated with almost all the soil properties. Among different indexes of soil properties, TP, AK has an important influence on the relative abundance of Motierellomycota, Chytridiomycota and Ascomycota, respectively.

## Discussion

### Differences in soil microbial community abundance and diversity between mixed *C. lanceolata*-broadleaf forest and pure *C. lanceolata* forest

Alpha diversity analysis of soil microbial community in the mixed *C. lanceolata*-broadleaf forest and the pure *C. lanceolata* forest revealed significant differences in microbial community diversity across different soil depth. Specifically, while Chao1 index indicated no significant differences in bacterial community richness in different soil depth under two forest stands, both Shannon and Simpson indices showed significantly higher bacterial community diversity in PC20 compared with PC40. This suggests that topsoil provides more favorable environmental conditions for bacterial community, such as greater organic matter input and better oxygen exchange conditions (Fierer et al., 2009). Meanwhile, the Simpson index was significantly higher in MC40 compared with PC40, while no differences were found between MC20 and MC40 on the alpha diversity of bacterial community. PCoA results based on Bray-Curtis distances showed substantial differences in bacterial community composition among different forest stands in different soil depth, which is considered a direct reflection of microbial community adaptability under different forest types in different soil depth (Prescott and Grayston, 2013). Notably, although there was no significant difference in beta diversity of bacterial community between different soil depth under the mixed forest stand, there was a significant difference in the beta diversity of bacterial community between the mixed forest and the pure forest at different soil depth, possibly due to increased environmental heterogeneity caused by main tree species diversity and differences in root activity (Van der Heijden et al., 2008). Mixed forest typically has higher ecosystem diversity and structural complexity, including different species of main trees, providing different ecological niches and resources for bacteria, thus supporting more diverse bacterial communities (Johnson and Swan, 2014). As for fungal community, although Shannon and Simpson indices were relatively lower in MC20 compared with PC20, topsoil showed significant differences in beta diversity of fungal community between PC20 and MC20. This indicates that the effects of soil depth and forest type on fungal communities are complex and may be related to different responses of fungi to soil chemical properties under different types of forest stand (Wardle et al., 2004). Meanwhile, the lower stand density of the pure forest stand might enhance the diversity of fungal community (Fernandez et al., 2024). Moreover, variations in soil chemical properties may affect the relative abundance of species with specific nutritional strategies within the fungal community (such as wood decay fungi and mycorrhizal fungi) (Allison and Martiny, 2008). Overall, these results highlight the importance of main tree species composition in shaping the structure and function of soil microbial communities in different soil depth. Different tree species affect different soil properties, such as pH, organic matter content, moisture, temperature and nutrient status, these factors directly or indirectly affect the diversity of soil microbial communities (Zhang et al., 2022). In summary, the mixed *C. lanceolata*-broadleaf forest have a more homogeneous bacterial and fungal communities in different soil depth compared with the pure *C. lanceolata* forest, wherein the mixed forest recruited more diverse bacterial community at subsoil and reduced the diversity of fungal community at topsoil.

### Differences in soil microbial community structure between mixed *C. lanceolata*-broadleaf forest and pure *C. lanceolata* forest

The microbial composition and abundance in different soil depth of the mixed *C. lanceolata*-broadleaf forest and the pure *C. lanceolata* forest exhibit significant differences. These variations may be influenced by multiple factors, such as plant root exudates, soil organic matter content, soil moisture, and other soil properties. The changes in the dominant phyla of bacteria and fungi under different forest stands in different soil depth illustrated that some specific microbiomes were selectively recruited at different soil depths with different tree species compositions (Jiao et al., 2018; Santoyo, 2022). For instance, Acidobacteriota and Proteobacteria, commonly found in soil environments, their high abundance suggests they play a crucial role in the nutrient cycle of the soil (Fierer et al., 2012a). The increased abundance of Chloroflexi in subsoil may reflect their adaptation to the unique environmental conditions of subsoil, consistent with previous studies showing Chloroflexi being more commonly found nutrient-poor environments (Speirs et al., 2019). Microbial biomarkers identified through differential species analysis indicate selective effects of different forest types and soil depths on specific microbial communities, which may be related to rhizosphere effects and plant species selection (Berendsen et al., 2012). Significant differences in bacterial biomarkers between MC and PC forest types suggest that these forest types may influence soil microbial community structure through root exudates or changes in soil chemical properties (Uroz et al., 2011). The variations in the dominant fungal phyla Ascomycota and Basidiomycota are also intriguing, as fungi from these phyla are commonly associated with nutrient cycling and organic matter decomposition in ecosystems (Lindahl and Tunlid, 2015). A higher abundance of Mortierellomycota in pure forests may indicate the phosphorus requirement of *C. lanceolata* rely on members of the phylum Mortierellomycota (Zhu et al., 2022). Overall, the differences of soil microbial community between the mixed *C. lanceolata*-broadleaf forests and the pure *C. lanceolata* not only reveals aspects of biodiversity but also provides key insights into the functioning and stability of soil ecosystems (Philippot et al., 2010). The mixed *C. lanceolata*-broadleaf forest might rely more diverse bacterial communities to make soil more stable and reduce nutrient loss, while the pure *C. lanceolata* might rely on more diverse fungal communities to meet their nutrient requirement.

The construction and analysis of bacterial and fungal co-occurrence networks at different soil depths reveal the complexity of microbial community structures and their differential responses to environmental disturbances. Bacterial co-occurrence networks in topsoil display higher stability than those in subsoil, possibly due to topsoil being more significantly affected by external factors such as temperature, moisture, and organic matter input, which may prompt bacteria in topsoil to form closer mutualistic networks to jointly withstand environmental changes. In contrast, fungal communities demonstrate higher network stability subsoil. This finding aligns with the research by Fuhrman (2009), which suggests that microorganisms in deeper soils are less affected by external disturbances and therefore may develop more robust network structures. As for different forest types, bacterial community co-occurrence networks in MC stands are more stable than those in PC stands, which may reflect the impact of different forest environmental conditions, such as main tree species diversity, organic carbon content, and soil pH values, on microbial community structure. Previous research has shown that the composition of main plant species can significantly affect soil microbial diversity and function (Venter et al., 2016), which may be a potential reason for the higher stability in MC stands. Additionally, in contrast to bacterial networks, fungal networks in PC stands have higher stability, which could be related to fungal high reliance on the decomposition of some specific organic matter in the soil, as fungi obtain nutrients through the decomposition of plant residues, and PC stands may provide more stable coniferous litter to support their recruiting specific fungal community. Waldrop et al. (2000) highlighted the role of fungi in decomposing forest floor residues, while Lindahl et al. (2007) demonstrated the coniferous forest maintain a stable fungal community to decompose needle litter and mobilize nutrient from decomposed organic matter. These studies confirm the importance of fungi in maintaining nutrient cycling and soil health in coniferous forest ecosystems, thereby the pure *C. lanceolata* forest need a more stable fungal community. Finally, the differences in network stability also reflect the resilience and recovery of different microbial communities to environmental disturbances. The stability of bacterial co-occurrence network in soil of both stands was higher in topsoil than that in subsoil. However, as for different stands, the stability of bacterial community co-occurrence network in mixed forest was higher than that in pure forest, indicating that soil bacterial community in mixed forest had better anti-interference ability (Wu et al., 2021).

### Relationship between soil microbial communities and soil chemical properties in mixed *C. lanceolata*-broadleaf forest and pure *C. lanceolata* forest

Firstly, a comparison between different soil depth revealed that, in mixed *C. lanceolata*-broadleaf forest, the contents of soil microbial biomass carbon (SMBC), soil microbial biomass nitrogen (SMBN), soil organic carbon (SOC), total nitrogen (TN), available nitrogen (AN), and available phosphorus (AP) in the topsoil are significantly higher than those in subsoil. A similar trend was observed in pure *C. lanceolata* forest for same soil indicators and AK. This could be attributed to the greater organic matter input and more active microbial activity in topsoil, which promotes the accumulation of microbial biomass and soil nutrients (Brady et al., 2008; Smith and Smith, 2011). As for different forest stands, it is evident that the contents of SMBC, SMBN, pH, and TP in the MC are significantly higher than those in the PC, suggesting that the mixed forest may provide more favorable environmental conditions that support microbial activity as well as the accumulation of microbial carbon, nitrogen and TP. However, when comparing soil layers at different depths (MC20 vs. PC20 and MC40 vs. PC40), only AP is significantly higher in the PC20 layer than that of MC20 layer, while there are no significant differences between MC40 and PC40 layers for SOC, TN, TK, and AN. This may indicate that the influence of tree species on these soil chemical properties diminishes at deeper soil depths (Wardle et al., 2004). When analyzing the relationship between microbial communities and soil chemical properties, Mantel's tests reveal a significant correlation between bacterial communities and MBC, MBN (*P* < 0.05), and a highly significant correlation with pH, SOM, SOC, TN, TP, AN, OP, AK (*P* < 0.01). This reveals a strong association between soil bacterial community structure and soil nutritional status (Fierer et al., 2012b). In contrast to bacterial community, fungal community only significantly correlates with TP and pH, which may suggest that fungal community has a specific role in soil phosphorus cycle and respond less sensitively to other nutrients compared with bacterial community (Treseder and Allen, 2022). Further exploration of the correlations between different microbial phyla and soil nutrient indicators reveals that within bacteria, Chloroflexi show negative correlations with most soil properties, as it was commonly found nutrient-poor environments (Speirs et al., 2019), enriched in nutrient-poor subsoil of the pure *C. lanceolata* forest. However, Verrucomicrobiota shows a positive correlation with all soil properties, members of which play pivotal role in biodegradation of complex chemicals and promote plants growth by mineralizing soil and thus enhancing soil nutrient level (Baliyarsingh et al., 2022), enriched in nutrient-rich topsoil of the mixed forest. This implies that different forest stands recruit different bacterial phyla to facilitate their soil nutrient cycle. The soil microbial biomass and nutrient level in mixed forest are generally higher than those in pure forest, especially in topsoil. Moreover, significant correlations exist between bacterial communities and soil nutritional level, while fungal community is only related to the soil phosphorus cycle, as the available phosphorus was higher in topsoil of the pure forest than mixed forest. These results suggested that the *C. lanceolata*-broadleaf forest rely on their specific bacterial community to mineralizing topsoil and thus maintain the high nutrient level, while the pure *C. lanceolata* forest still rely on some specific fungi, i.e., Sordariomycetes and Agaricales being involving the phosphorus cycle as ectomycorrhizal fungi, to meet their phosphorus requirement, as the available phosphorus is limited in South China.

## Conclusions

This study indicates significant differences in soil chemical properties, microbial composition, and microbial diversity in different soil depths (0–20 cm and 20–40 cm) between different forest stands (mixed *Cunninghamia lanceolata*-broadleaf forest and pure *C. lanceolata* forest). In summary, the topsoil layer exhibited significantly higher values in soil nutrient level compared with the subsoil layer under both forest stands. In terms of soil microbial community, the mixed *C. lanceolata*-broadleaf forest had a more homogeneous bacterial and fungal communities at different soil depth compared with the pure *C. lanceolata* forest, wherein the mixed forest recruited more diverse bacterial community in subsoil and reduced the diversity of fungal community in topsoil. Simultaneously, the bacterial network stability in topsoil is greater than in subsoil, and the bacterial network stability in mixed forests exceeds that in pure *C. lanceolata* forests. Conversely, the fungal network stability in subsoil is higher than in topsoil, with the stability of fungal networks in pure *C. lanceolata* forests surpassing that in mixed forests. Regarding the relationship between microbial communities and soil chemical properties, bacterial community showed significant correlations with various soil chemical indicators, whereas fungal communities exhibited correlations with only the TP and pH. Therefore, different strategies were maintained in different soil depth of mixed *C. lanceolata*-broadleaf forest and pure *C. lanceolata* forest, respectively, i.e., the mixed *C. lanceolata*-broadleaf forest rely on their recruiting bacterial community to enhance and maintain the higher nutrient status of soil while the pure *C. lanceolata* forest rely on some specific fungi to satisfy their phosphorus requirement for survive strategy. In future, it is worth to try a new strategy in modifying the soil fertility of pure *C. lanceolata* forest by applying some soils from the mixed *C. lanceolata*-broadleaf forest as microbial fertilizer.

## Data availability statement

The datasets presented in this study can be found in online repositories. The names of the repository/repositories and accession number(s) can be found in the article/supplementary material.

## Author contributions

FZ: Conceptualization, Investigation, Software, Writing – original draft, Writing – review & editing. JG: Investigation, Writing – original draft. DL: Investigation, Software, Writing – original draft. JY: Investigation, Writing – original draft. XS: Investigation, Writing – original draft. CL: Investigation, Writing – original draft. HC: Funding acquisition, Investigation, Supervision, Writing – original draft, Writing – review & editing.
